# Synopsis of Meeting of Virginia State Veterinary Medical Association

**Published:** 1897-08

**Authors:** 


### SOCIETY PROCEEDINGS.

### SYNOPSIS OF MEETING OF VIRGINIA STATE VETERINARY MEDICAL ASSOCIATION.

THE fourth annual meeting was called to order by the President, Dr. George C. Faville, in the office of Dr. W. H. Harbaugh, at Richmond, Va., on Wednesday, June 23, 1897.

After the roll-call the President addressed the Association as follows: "Another year has rolled around, and it becomes your duty to elect those officers who shall have the welfare of your organization in their keeping for one more year.

"The activity displayed during the first two years of our existence as an organization makes the last year seem, by comparison, like a vacation. Considerable has been accomplished by many of our members working in individual lines. Much yet remains undone.

"The professional standing of our Association, and the standing of each member individually, depend upon our professional activity largely, and largely upon our desires. That the educated veterinarian should stand upon the same footing socially, professionally, and every other way is axiomatic.

That in many places he does is a fact; but in Virginia, unfortunately, he too often is placed, by those employing him, upon the same footing as the blacksmith, the old-time horse-doctor, and the charlatan. This is largely due to the veterinarian himself. Too frequently he is wiling to be put upon the lower plane in order to secure the case. Too often he submits to an unworthy competition in order not to make enemies, and too often he is willing to think that when he has his diploma he has all that can be gotten of veterinary knowledge, and is ready to settle down into a jog-trot of professional practice, making no adequate effort to keep up with the procession. To keep in the vanguard requires continued effort. How continual only those who try hard to keep there can tell.

"Much good work has been done during the last year. The work done by our worthy member, Dr. Harbaugh (notwithstanding much sickness), in his researches into the milk-supply of our capital city is an example to the rest of us. His work must result in much good, and command the attention of the whole medical fraternity. The lack of knowledge, worse than all, the lack of the desire for information regarding the milk-supply of our cities, is a surprise to all those who look into the question. Political boards of health and city councils, whose only care is to cater to the wishes of the ward-boss, and who will not look into the merits of the question of proper food-supplies, are a menace to the heathfulness of our cities. There is not a city in our State that does not control the running at large of dogs, because of danger to the health of the public from a largely imaginary trouble—rabies; or which does not compel a careful sanitary inspection of all houses within its limits in order to prevent the propagation of disease, or which does not give its board of health control of many conditions which are deleterious to health; but this one, most important of all—the milk-supply—is not considered, although the most dangerous of all from many considerations. Our Association has accompished much, but much remains to be done."

The report of the Board of Censors recommended the election to membership in the Association of Dr. H. S. Willis, of Rapidan Station, Va., which report was accepted, and Dr. Willis was accordingly elected to membership. The resignations of Drs. Marshall and Dixon, which were tendered on account of their removal from the State, were accepted.

On motion of Dr. Harbaugh, which was seconded and passed, the semi-annual meetings were discontinued for the present. Dr. Harbaugh was also author of the motion that the President appoint two essayists to read papers at the next regular meeting.

After receiving the reports of the committees and transacting other routine business, the election of officers took place, which resulted in the reelection of the following officers: President, Dr. George C. Faville, Norfolk; First Vice-President, Dr. W. T. Gilchrist, Norfolk; Second Vice-President, Dr. Charles McCulloch, Howardsville; Secretary, Dr. Thomas M. Sweeny, Richmond; Treasurer, Dr. A. J. Burkholder, Staunton. The President appointed as members of the Board of Censors, Drs. Harbaugh, Roop, Willis, Drake, and Niles. Committee on Statistics, Drs. Harbaugh (chairman), Gilchrist, and Roop. Committee on Jurisprudence and Legislation, Drs. Harbaugh (chairman), Niles, and Sweeny.

**Remember the dates, 7th, 8th, and 9th of September.**

The afternoon was devoted to the reading and discussion of reports of cases, after which the Association adjourned to meet in Norfolk on the fourth Wednesday of June, 1898.

THOMAS M. SWEENY,
Secretary.

REPORT OF THE COMMITTEE ON STATISTICS.

You will observe that as Chairman of this committee I have the same complaint to make as my predecessor. I cannot understand why members neglect to send in notes upon such an important matter when the benefits are mutual.

Dr. E. P. Niles, State Veterinarian, sends the following report, which I include without change.

"Pursuant to your call for notes on contagious epizootic and enzootic diseases, I beg to submit the following:

"Since the last report of your committee I have observed encephalitis in various parts of the State. This is a disease which gives rise to many 'mad-dog' scares, and I have seen it in horses, mules, cattle, and dogs. Last September the disease was prevalent in Northampton County among mules and horses. The symptoms vary somewhat in the different animals and even in animals of the same species. In the horse and mule, in cases which have come under my observation, the 'rabid' symptoms have been less marked than in cattle and dogs. The temperature has remained nearly normal; appetite not impaired, at least in the early stage of the disease, the animal showing a desire to partake of food and water, but unable to do so on account of the paralysis of the muscles of the throat. In the case of a mule to which my attention was called, the paralysis of the throat was, at that time, the only symptom of disease. Cattle usually become very excitable, and frequently vicious, attacking anything which may come in their way, especially dogs. At first the symptoms are mild, and to the ordinary observer not well defined, but upon examination it is noticed that the animal shows a peculiar indifference to its surroundings, there is a characteristic staring appearance of the eyes, and, owing to the partial or complete paralysis of the throat, the saliva dribbles from the mouth. As the disease advances the animal becomes more easily excited, and if not confined may wander about in an aimless manner, frequently stopping and bellowing for several minutes at a time; if other animals come its way it makes a vicious charge upon them. In advanced stages of the disease the spinal cord may also be involved, causing partial and sometimes total paralysis of the extremities, manifested by a staggering gait or inability to stand. The true cause of this disease seems not to be well understood. I am of the opinion, however, that it is an infectious disease due to a fungus. In one outbreak, which I have investigated, the disease was confined to a certain valley through which a stream of water ran. On this stream of water are located several sawmills, the sawdust from which is thrown into the stream. I am of the opinion that the decaying sawdust in this stream had something to do in the causation of the disease in this instance.

**Boston wants the meeting in 1898.**

"Canine distemper has been more prevalent in this section of the State during the past two months than at any time since I have resided here. Some of the cases are of a very mild nature, while others are very severe.

"Enzoötic pharyngitis made its appearance in this section during the spring for the first time; several cases have been brought to the clinics held by Dr. Roop at the college veterinary hospital. The most of such cases have yielded very promptly to treatment, complications arising only in one case. In that case it became necessary to trephine the inferior maxillary sinus to remove the pus which had collected there.

Distemper has also been prevalent during the past three months in a much more severe form than usual.

"A few cases of black-leg have also been reported. This disease has, however, been confined to a very limited area.

"Splenetic or Texas cattle-fever prevailed in certain sections of the State during last summer, but since the quarantine rules and regulations have been in force but two outbreaks of the disease occurred above the quarantine line."

I am pleased to announce that Dr Niles has succeeded in having a Board of Health established in the town of Blacksburg, consisting of one veterinarian and two physicians. He also induced the town council to pass an ordinance, which is now in force, requiring meat- and milk-inspection for the town, this being the first and only place in the State having such a law.

Dr. H. Bannister, of Roanoke, reports as follows:

"During the year past I have had considerable of a sort of meningitis that appears to be infectious to some extent; it has occurred mostly in cows, calves, and some in dogs. The symptoms in the main are identical, excepting that in adult cattle there appears to be more violence than in either dogs or calves. Treatment has, in my practice, been of no avail, death usually occurring in from thirty-six to forty-eight hours. I have seen but two recoveries, both in cattle, one a four-year-old Jersey, and the other a yearling. The cow was given small doses (ʒij) of chloral hydrate, and the heifer got no treatment.

"A very mild form of pharyngitis has been very prevalent this year, requiring very little treatment, and, beyond the annoyance of incessant coughing, giving no trouble.

"Strangles in light form has occurred as it usually does."

Dr. H. S. Drake, of Leesburg, sends a very important report, and I fail to see how we can overlook some parts of it. There can be no question that the Association should investigate as he suggests. His report is as follows:

"Tuberculosis is prevalent in every dairy of the county to a greater or less degree. Nothing is being done to check its spread, except in some one or two instances where I have examined by physical means, in order to destroy or remove all diseased cattle before new ones are brought in for renewing the dairy. Others have been warned, but no notice is being taken of the law. Many do not know that there is any danger. I do not think that the medical doctors are giving warning to their patrons, as I know of the supply for babies being furnished from cows that have been diseaed for some years, and the M.D.'s of the town are surely aware of the existence of the disease.

**Special railroad rates from all the large cities.**

"Osteoporosis has been observed in a number of horses boarding at Oak Hill Farm, that are natives of Pennsylvania, near Philadelphia. They were diseased when brought here about one year ago. Some have died, and others were destroyed at my instigation, where fractures existed. I now have two geldings, three years old, and two brood-mares under observation. Notes as follows: September 17, 1895, two colts, sorrel and black, two years old. Enlargement of superior maxillary in both very bad. Gait retarded and stiff, otherwise in good condition. Emasculation standing. Both did well. I was informed by Mr. A. (owner) that the animals were getting much better, as the enlargement is scarcely perceptible to ordinary observers. I saw them on May 26, 1897, and would not have believed there could be such a change in so short a time, and without treatment. Their movement is free, and with a new vigor of life, as is clearly demonstrated by their ability to kick and jump. The two brood-mares, as stated by Mr. F., lost their foals before coming to his farm, and they are perceptibly diseased, but have by their side two fine foals as ever grew, and apparently sound at this writing. I hope to get a full history of the mares, and to keep track of their offspring as developed, and note results for further reference.

**The South summons YOU to their support.**

"My attention has been called at different periods to fistulæ of withers and poll existing among so many horses of all classes and types. I wish to bring before the profession the advisability of investigation by the able professional staff of our State institute. It is causing the loss of many valuable animals in this and adjoining counties. It is more prevalent on the dairy-farms of this county and among young, unbroken colts. All cases can be traced to there having been some affected animal on the premises before the outbreak, and in many places every animal has been affected at different periods of time, and when all horses have been removed from the farm, and a new tenant comes with stock free from any such disease, in four to six months there will be from one to three animals showing swelling either of withers or poll. As to forms, I have noted that one may be classed as a fibrous enlargement, the other of sero-purulent type, which, in my experience, is the more common, and I believe it to be of a contagious or infectious nature, while the fibrous form I do not consider to be so. The fibrous form has a number of ducts, while the other form has but a single duct connecting directly with the spinous processes or the shoulder and its integuments."

In Richmond and vicinity during the past year, muscular rheumatism has prevailed to more than the average extent.

Pharyngitis among horses, which first made its appearance here several years ago in a mild form, now appears in many instances as a malignant disease, and, in fact, all cases of it require closer attention than formerly.

About the average number of cases of tetanus have occurred.

The nervous form of distemper in the dog has caused a large death-rate.

Encephalitis of cattle seems to have moved to the western part of the State. I was called to attend a few isolated cases during the year, but I heard of no outbreak of any importance, such as we had a few years ago.

Respectfully,

W. H. HARBAUGH,
Chairman.

